# PyAMPA: a high-throughput prediction and optimization tool for antimicrobial peptides

**DOI:** 10.1128/msystems.01358-23

**Published:** 2024-06-27

**Authors:** Marc Ramos-Llorens, Roberto Bello-Madruga, Javier Valle, David Andreu, Marc Torrent

**Affiliations:** 1 Systems Biology of Infection Lab, Department of Biochemistry and Molecular Biology, Biosciences Faculty, Universitat Autònoma de Barcelona, Barcelona, Spain; 2 Barcelona Biomedical Research Park, Department of Medicine and Life Sciences, Universitat Pompeu Fabra, Barcelona, Spain; London School of Hygiene & Tropical Medicine, London, United Kingdom

**Keywords:** antimicrobial peptide

## Abstract

The alarming rise of antibiotic-resistant bacterial infections is driving efforts to develop alternatives to conventional antibiotics. In this context, antimicrobial peptides (AMPs) have emerged as promising candidates for their ability to target a broad range of microorganisms. However, the development of AMPs with optimal potency, selectivity, and/or stability profiles remains a challenge. To address it, computational tools for predicting AMP properties and designing novel peptides have gained increasing attention. PyAMPA is a novel platform for AMP discovery. It consists of five modules, namely AMPScreen, AMPValidate, AMPSolve, AMPMutate, and AMPOptimize, that allow high-throughput proteome inspection, candidate screening, and optimization through point-mutation and genetic algorithms. The platform also offers additional tools for predicting and evaluating AMP properties, including antimicrobial and cytotoxic activity, and peptide half-life. By providing innovative and accessible inroads into AMP motifs in proteomes, PyAMPA will enable advances in AMP development and potential translation into clinically useful molecules. PyAMPA is available at: https://github.com/SysBioUAB/PyAMPA

**IMPORTANCE:**

This paper introduces PyAMPA, a new bioinformatics platform designed for the discovery and optimization of antimicrobial peptides (AMPs). It addresses the urgent need for new antimicrobials due to the rise of antibiotic-resistant infections. PyAMPA, with its five predictive modules -AMPScreen, AMPValidate, AMPSolve, AMPMutate and AMPOptimize, enables high-throughput screening of proteomes to identify potential AMP motifs and optimize them for clinical use. Its unique approach, combining prediction, design, and optimization tools, makes PyAMPA a robust solution for developing new AMP-based therapies, offering a significant advance in combatting antibiotic resistance.

## INTRODUCTION

Antibiotic-resistant bacterial infections are a major global health threat, as current treatments are often ineffective against life-threatening strains, highlighting the urgent need for new strategies to treat bacterial infections (1–6). One promising approach is the use of antimicrobial peptides (AMPs), which have emerged as a potential alternative to conventional antibiotics (7). AMPs are naturally occurring molecules that can disrupt bacterial membranes or modulate immune responses, making them promising candidates for therapeutic development (8, 9).

Computational approaches to inspect proteins in search of regions with antimicrobial activity have spurred the development of novel software capable of predicting AMP motifs with high accuracy (10–15). Advantages of such computational approaches (16) include the ability to screen vast protein data in search of potential AMP sequences, well beyond the reach of any experimental approach (17–19), plus the complementary ability to predict structural and/or physicochemical features leading to effective AMP candidates (9, 18, 19).

The AMPA server (10), our most relevant contribution so far to such computational tools, uses a sliding-window approach to identify sequences with potential antimicrobial activity within a protein, and has been successfully used by us and other groups (20–24) to propose and evaluate novel AMP leads. The scope of the original AMPA, however, was somehow limited in that it was primarily implemented to screen one or just a small set of protein sequences, and did not provide any other useful information (e.g., toxicity and stability) on the predicted AMP motif.

In this paper, we introduce PyAMPA as a fully revamped version of AMPA where the original features are improved by machine-learning modules capable of predicting effective AMP motifs with low toxicity and enhanced serum half-life. To demonstrate experimentally the PyAMPA fitness, we identify several novel AMP leads with broad and potent activity against a diverse panel of bacterial strains. Overall, PyAMPA constitutes a valuable, cost-effective tool for developing AMP-based therapies in the context of the global antibiotic resistance crisis.

## MATERIALS AND METHODS

### Data sets and data curation

AMPs were retrieved from the AMPlify data set of experimentally validated AMPs (12, 13). The data set contains 6,676 peptides, evenly split into 3,338 AMPs and 3,338 non-AMPs. For cell-penetrating peptides (CPPs), the Liu et al. database (25) was used, containing 924 sequences, of which 462 positive and 462 negative CPPs. To predict hemolytic peptides, we used the AMPDeep database (26) of 7,180 peptides, of which 3,007 hemolytic and 4,173 non-hemolytic. Similarly, for toxicity prediction, we used the ToxinPred database (27), with 3,613 peptides, of which 1,156 toxic and 2,457 non-toxic. For all databases, peptides shorter than seven residues or containing modified residues were excluded. In all data sets, no significant differences in length distributions between active and inactive classes were observed (Fig. S1). Finally, to predict activity in different bacterial species, IC_50_ data were retrieved from the *Giant Repository of AMP Activities* (GRAMPA) (28) (https://github.com/zswitten/Antimicrobial-Peptides). Species in the GRAMPA database include *Escherichia coli*, *Staphylococcus aureus*, *Pseudomonas aeruginosa*, *Candida albicans*, *Bacillus subtilis*, *Staphylococcus epidermidis*, *Salmonella typhimurium*, *Micrococcus luteus*, *Klebsiella pneumoniae*, *Enterococcus faecalis,* and *Acinetobacter baumannii*.

### PyAMPA modules

**Fig 1:**
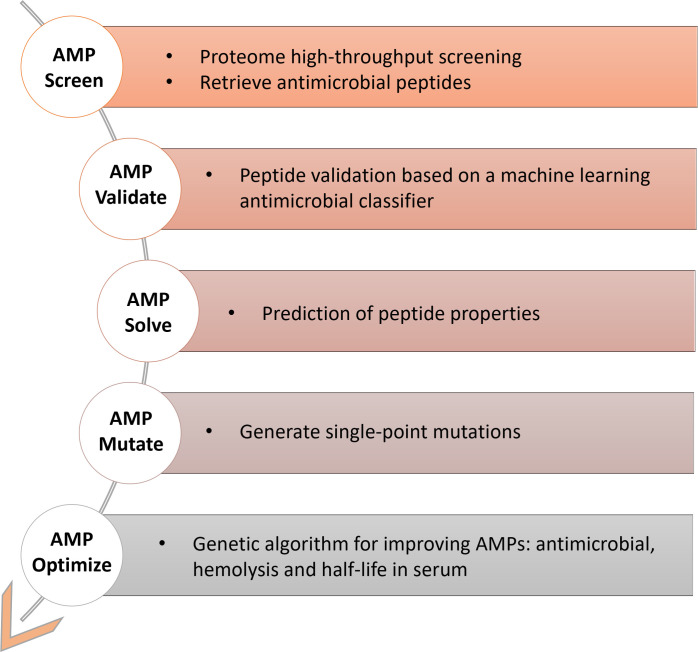
Workflow of PyAMPA. AMP discovery and development modules in proteome high-throughput screening for initial candidates (AMPScreen and AMPValidate), property prediction (AMPSolve), and subsequent optimization toward biomedical application (AMPMutate and AMPOptimize).

In PyAMPA, several modules are integrated to predict and optimize novel AMPs (Fig. 1). In the first stage, AMPAScreen works as a high-throughput predictor of antimicrobial regions, even entire proteomes. After this initial screen, peptides are further processed using AMPValidate, an algorithm based on a vectorizer model to validate active peptides. Finally, AMP features most relevant for future development, namely hemolysis, toxicity, serum half-life, cell-penetrating capacity. and antimicrobial activity against a broad panel of microorganisms, are evaluated by the AMPSolve module. Candidates can be further assessed by AMPMutate, an algorithm identifying possible point mutations hence key residues in AMPs. Finally, the most relevant features can be additionally refined using the AMPOptimize module.

#### AMPScreen: a high throughput antimicrobial peptide predictor

AMPScreen is a new implementation of an antimicrobial propensity scale first described by Torrent et al. (29), and used in the original AMPA software (10). While effective in identifying antimicrobial regions in proteins, AMPA has limited ability to generate high-throughput predictions. In contrast, AMPScreen enables the processing of entire proteomes. As before, AMPScreen reads each protein entry sequentially and employs a sliding window screen where each sequence position is scored as the average of residues in the window (default window size of 7).

To discriminate antimicrobial from non-antimicrobial regions, a propensity threshold is established. For each position at the center of the windowed sequence, if the average score is below the threshold, the position is labeled as antimicrobial (1); otherwise, it is labeled as non-antimicrobial (0). For a sequence to be considered a potential AMP motif, it must comprise an array of at least 12 consecutive positive values, with only two gaps allowed. While an optimal default threshold of 0.225 [as in the original AMPA; (10)] is suggested, users can modify both the window size and the threshold value in the AMPScreen script.

#### AMPValidate: a random forest classifier for validating AMPs

To further refine the results, PyAMPA includes a filter called AMPValidate, based on a random forest classifier. First, peptide sequences in the training database are pre-processed and a vectorizer is used to convert them into a numerical format that can be used by machine learning algorithms. A vectorizer tokenizes a text, meaning it splits it into individual “tokens” or “terms.” In AMPValidate, these tokens are dipeptide (2-mer) units of the global sequence. Once the text is tokenized, the vectorizer builds a vocabulary, which is essentially a mapping of each unique token to a unique integer index. This vocabulary contains all the unique tokens present in the entire data set. Hence, for each sequence, the vectorizer counts how many times each token from the vocabulary appears in that sequence. These values are then used to build a random forest classifier. The characteristics programmed for the random forest classifier were an arrangement of 250 trees in the forest, with a minimum number of 1 leaf per tree and an equal number of weights per leaf node.

#### AMPSolve: a prediction tool for peptide hemolysis, toxicity, half-life, and antimicrobial spectrum

All models included in AMPSolve were developed using random forest classifiers, except for peptide half-life. For hemolysis and toxicity, properties were predicted by random forest classifiers using the same strategy as described for AMPValidate. For the antimicrobial spectrum, a random forest regressor based on a 2-mer vectorizer was created for each strain in the database, including *E. coli*, *S. aureus*, *P. aeruginosa*, *C. albicans*, *B. subtilis*, *S. epidermidis*, *S. typhimurium*, *M. luteus*, *K. pneumoniae*, *E. faecalis*, and *A. baumannii*.

To predict peptide half-life, PyAMPA uses estimations based on a multivariable regression model developed by Cavaco et al. (30). The equation that describes the regression model for estimating half-life in minutes depends on the presence of nonpolar amino acids, the presence or absence of Trp (W) and Tyr (Y), and on peptide isoelectric point (IP), as follows:


$$\ln\left(t_{\frac{1}{2}}\right)=\;\;2.226+\left(0.053\times NP\left[\%\right]\right)-\left(1.515\times W\left[0,1\right]\right)+\left(1.290\times Y\left[0,1\right]\right)-\left(1.052\times IP\left[0,1\right]\right)$$

where NP [%] is the percentage of nonpolar residues (Ala, Cys, Gly, Ile, Leu, Met, Phe, Pro, Trp, Tyr, and Val); W [0,1] is the absence (0) or presence of at least one Trp (1); Y [0,1] is the absence or presence of just one Tyr (0) or at least two Tyr (1), and IP [0,1] is IP < 10 (0) or IP > 10 (1).

#### AMPMutate: a peptide mutagenesis tool

To investigate comprehensively the effect of point mutations on candidate AMPs and their antimicrobial activity, we devised a straightforward algorithm that systematically replaces individual residues within the original peptide sequence. Each amino acid position in the peptide sequence is successively replaced with the 20 coded amino acids to generate all possible point mutations. The AMPMutate output contains all mutated peptides derived from the original sequence, including their antimicrobial and hemolytic probability, and estimated half-life. AMPMutate displays these results as heatmaps to assist users in identifying significant amino acid positions.

#### AMPOptimize: an AMP optimization tool

AMPOptmize is a genetic algorithm tool for generating novel AMPs with desirable clinical characteristics, including high antimicrobial activity, long half-life, and low hemolytic activity. Initially, a first generation of peptides (100 sequences) is created by random mutation and selected based on a fitness function defined as:


$$F=w_{A}\cdot P_{A}+w_{H}\cdot P_{H}+w_{HL}\cdot HL_{Norm}$$

where *P_A_* is the antimicrobial probability, *P_H_* is the hemolytic probability, and *HL_Norm_* is the predicted half-life, normalized in the range [0,1]. Each variable is weighted with the corresponding parameter (*w_A_*, *w_H_*, *w_HL_*) that can be adjusted depending on design preferences. In the original AMPOptimize, weights are set to 1 by default.

A subset (20%) of peptides with best fitness values are selected to replace the initial population of peptides (elitism). These peptides are then subjected to one-point crossings with each other, a process that involves selecting two sequences at random and performing a recombination step on a randomly selected position in the sequence (crossover rate 0.8, by default). In addition, peptides can undergo one-point mutations to create new sequences (mutation rate 0.2, by default). The properties of all peptides thus modified are again predicted and their fitness calculated as defined above. A first generation is created, and the algorithm is by default allowed to run for 100 generations or until no improvement in fitness score after 20 rounds is observed. The sequence reaching the highest fitness score is then selected and displayed. AMPOptimize also displays helical wheel projections of the original and modified sequences for the user to compare differences.

#### Validation

To validate PyAMPA modules, the data sets were split into 60% training, 20% validation, and 20% testing. The validation set is used during the training to prevent overfitting. Accuracy and Mathew’s correlation coefficient (MCC) were used as indicators of global performance.


$$\begin{array}{cc} & \text{ Accuracy }=\frac{TP+TN}{P+N}\\ & MCC=\frac{TP\times TN-FP\times FN}{\sqrt{\left(TP+FP)\times (TP+FN)\times (TN+FP)\times (TN+FN\right)}} \end{array}$$

where TP is true positive, TN is true negative, FP is false positive, FN is false negative, P is total positive entries, and *N* is the total number of negatives in the data set.

In addition, receiver operating characteristic (ROC) and precision-recall (PR) curves were analyzed for each module. The area under the ROC curve (AUROC) and area under the PR curve (AUPR) were used as an indicator of the classification performance of the system.

#### Implementation

PyAMPA was developed using Python 3.11. Python libraries used for coding were NumPy (1.24.3) for array computing, Pandas (2.0.2) for data analysis, SciKit-Learn (1.2.2) for machine learning, BioPython (1.81) for computational biology; and Matplotlib (3.7.1) and Seaborn (0.12.2) for plotting.

### Experimental validation

#### Peptide synthesis

Peptides (Table S1) were assembled as C-terminal carboxamides at 0.1 mmol scale on H-Rink amide ChemMatrix resin (PCAS BioMatrix, Quebec, Canada) on a Prelude instrument (Gyros Protein Technologies, Tucson, AZ) using Fmoc chemistry. Side-chains of trifunctional residues were protected with Boc (Lys, Trp), N^G^-2,2,4,6,7-pentamethyldihydrobenzofuran-5-sulfonyl (Arg), tert-butyl (Ser, Asp, Glu) and trityl (Asn, Cys) groups. Couplings used 8-fold molar excess of both Fmoc-amino acid and HBTU, in the presence of 16-fold molar excess of DIEA in N,N-dimethylformamide (DMF). Fmoc was removed with piperidine/DMF (20:80 vol/vol). After chain assembly, full deprotection and cleavage from the resin were performed with TFA/H_2_O/TIS/DODT (94:2.5:2.5:1 vol/vol, 90 min) at room temperature. Crude peptides were isolated from the TFA solution by precipitation with chilled diethyl ether and centrifugation at 5000 rpm, 2 × 10 min, 4°C, then dissolved in water and lyophilized.

Analytical RP-HPLC was done on an LC-2010 instrument (Shimadzu, Kyoto, Japan) using a Luna C18 column (4.6 × 50 mm, 3 µm; Phenomenex), eluted with a 0%–95% linear gradient of B (0.036% TFA in MeCN) into A (0.045% TFA in H_2_O) over 15 min at 1 mL/min flow rate, with UV detection at 220 nm. Peptides were purified by preparative RP-HPLC on a Shimadzu LC-20AP instrument, using an Aeris Peptide XB-C18 column (21.2 × 250 mm, 5 µm; Phenomenex), eluted with a 10%–50% linear gradient of B (0.1% TFA in MeCN) into A (0.1% TFA in H_2_O) over 30 min at 20 mL/min flow rate, UV detection at 220 nm. LC-MS was performed on a 2010 EV instrument (Shimadzu) fitted with an Aeris Widepore XB-C18 column (4.6 × 150 mm, 3.6 µm, Phenomenex), eluting with linear gradients of B (0.08% FA in ACN) into A (0.1% FA in H_2_O) over 15 min at 1 mL/min flow rate. Fractions with >98% HPLC homogeneity and the expected mass were pooled and lyophilized.

#### Minimum inhibitory concentration

Antimicrobial activities of all peptides were determined according to the methodology described for AMPs (31). The minimum inhibitory concentration (MIC) was defined as the lowest concentration of the test peptide at which microorganism growth was visibly absent. The assay was done in polypropylene 96-well plates that prevent peptide binding (Greiner, Frickenhausen, Germany). From a bacterial isolate, a colony was seeded in Mueller Hinton Broth (MHB) and allowed to grow at 37°C in an incubator with orbital shaking at 250 rpm overnight. Subsequently, a new bacterial culture was seeded from the overnight culture and allowed to grow to an OD of 0.5 at 600 nm. The bacterial suspension was diluted to a final virtual concentration of 5 × 10^5^ CFU/mL in MHB medium and was used as the working culture. Next, 20 µL of twofold serial dilutions of each peptide were dissolved in water containing 0.4% wt/vol bovine serum albumin (BSA) and 0.02% vol/vol glacial acetic acid to prevent self-aggregation, based on the classical microtiter broth dilution recommended by the National Committee of Laboratory Safety and Standards. Then, 80 µL of working culture was applied to each well. The plate was incubated for 24 h at 37°C. The results are the average of three independent experiments.

#### Hemolytic activity

The hemolytic activity of peptides was determined by detecting the disruption of horse red blood cells (RBCs), as described (32) . Briefly, fresh RBCs in PBS (pH 7.4) were first centrifuged 3 × 1,500 × *g* for 5 min, then diluted 1:10 with phosphate-buffered saline (PBS) to yield an RBCs concentration of ~5 × 10^8^ cells/mL. RBCs were then incubated for 1 h at 37°C with twofold serial dilutions of peptides covering a 0.24–250 μM concentration range. 1% Triton X-100 and PBS were used as positive and negative controls, respectively. After incubation, samples were centrifuged at 1,500 × *g* for 10 min at room temperature, and the supernatant (60 µL) was transferred to a flat-bottomed 96-well polypropylene microplate (Greiner, Frickenhausen, Germany). Hemoglobin release was then measured as OD at 540 nm in a Tecan Infinite microplate reader (Tecan, Männedorf, Switzerland). Percent hemolysis was calculated as indicated below. The results are the average of three independent replicates.


$$\%\;Hemolysis=\frac{\left[\;OD\;Peptide{\;}_{540}\right]-\left[OD\;PBS_{540}\right]}{\left[OD\;Triton_{540}\right]-\left[OD\;PBS_{540}\right]}\times 100$$

#### Cytotoxicity in mammalian cells

HepG2 cells were cultured in Eagle’s minimum essential medium (MEMα), supplemented with 10% (vol/vol) fetal bovine serum (FBS) and 1% penicillin (100 IU/mL)/streptomycin (100 µg/mL) solution, and maintained at 37°C in a humidified atmosphere with 5% of CO_2_. After cells were grown, they were seeded into 96-well plates at 3 × 10^4^ cells/well and cultured overnight to adhere to the plate. Subsequently, adherent cells were incubated for 4 h under standard conditions (5% CO_2_, 37°C) with twofold serial dilutions of each peptide in a 1.56–200 μM concentration range. Following incubation, the peptide-containing medium was replaced with MEM supplemented with FBS and 3-(4,5-dimethylthiazol-2-yl)-2,5 diphenyltetrazolium bromide (MTT) at 0.4 mg/mL and the cells were further incubated for 120 min. Formazan crystals in live cells were detected after disruption with 200 µL of DMSO by OD measurement at 570 nm in a Tecan Infinite microplate reader. 1% Triton X-100 and PBS were used as positive and negative controls, respectively. Data are the average of three independent replicates.

#### Circular dichroism spectroscopy

Circular dichroism (CD) experiments were performed at room temperature on a J-815 spectropolarimeter (Jasco, Tokyo, Japan) using quartz cells of 1.0 mm thickness. Peptides were dissolved in H_2_O and in a 1–30 mM SDS concentration range to simulate a membrane environment at a final 50 µM concentration in all cases. Spectra were recorded in the 190–250 nm range and accumulated 15× to improve the signal-to-noise ratio. Baselines of samples without peptides in either water or micelle suspension were subtracted from each reading to calculate the peptide contribution. Primary data were processed in OriginPro 2022, with the Savitzky-Golay method used for smoothing. The molar mean residue ellipticity [*θ*] was calculated as follows:


$$\left[\theta \right]\;\left(\deg cm^{2}\;dmol^{-1}\right)=\;\;\frac{\theta \left(mdeg\right)}{10\times \;L\times \;\left[M\right]\times \;N}$$

where *L* is the path length (cm), [*M*] is the concentration (mol/L), and *N* is the number of peptide bonds, *i.e*., the number of residues minus one.

## RESULTS AND DISCUSSION

### Description of PyAMPA

PyAMPA is a new software based on AMPA, a prediction tool originally designed to identify antimicrobial regions in proteins. While the previous version of AMPA can successfully define “antimicrobial pharmacophores” of proteins, it is unsuitable for massive screenings, predicting peptide properties or optimizing structures. In this work, we present PyAMPA, a much upgraded version of AMPA designed to screen large protein data sets quickly and accurately.

Most AMP prediction tools focus solely on antimicrobial activity. However, high activity does not guarantee a successful AMP. Other factors, such as stability and toxicity, must also be considered. Furthermore, some peptides may target either Gram-negative or Gram-positive bacteria, resulting in different antimicrobial profiles. PyAMPA is equipped with several modules that estimate all these properties and provide an educated guess on whether an AMP is suitable for laboratory testing (Fig. 1). PyAMPA first runs its AMPScreen module to quickly locate potential antimicrobial regions in a large data set. Once the potential peptides are identified, the AMPValidate module further distinguishes “proper” AMPs from other peptides. For validated peptides, an AMPSolve module predicts properties such as cell-penetrating capacity, hemolytic activity, toxicity, serum half-life, and antimicrobial profile against several bacterial species. An ideal AMP should have broad and potent antimicrobial activity, low toxicity, and a long half-life, and AMPSolve enables the user to select peptides with such features for specific purposes. PyAMPA also includes two additional modules, AMPMutate and AMPOptimize, that can further fine-tune the sequence for improved properties. AMPMutate predicts the properties of all single-point mutations in the peptide, while AMPOptimize uses a genetic algorithm to enhance several peptide properties.

### Screening and validating AMPs using AMPScreen and AMPValidate

**Fig 2:**
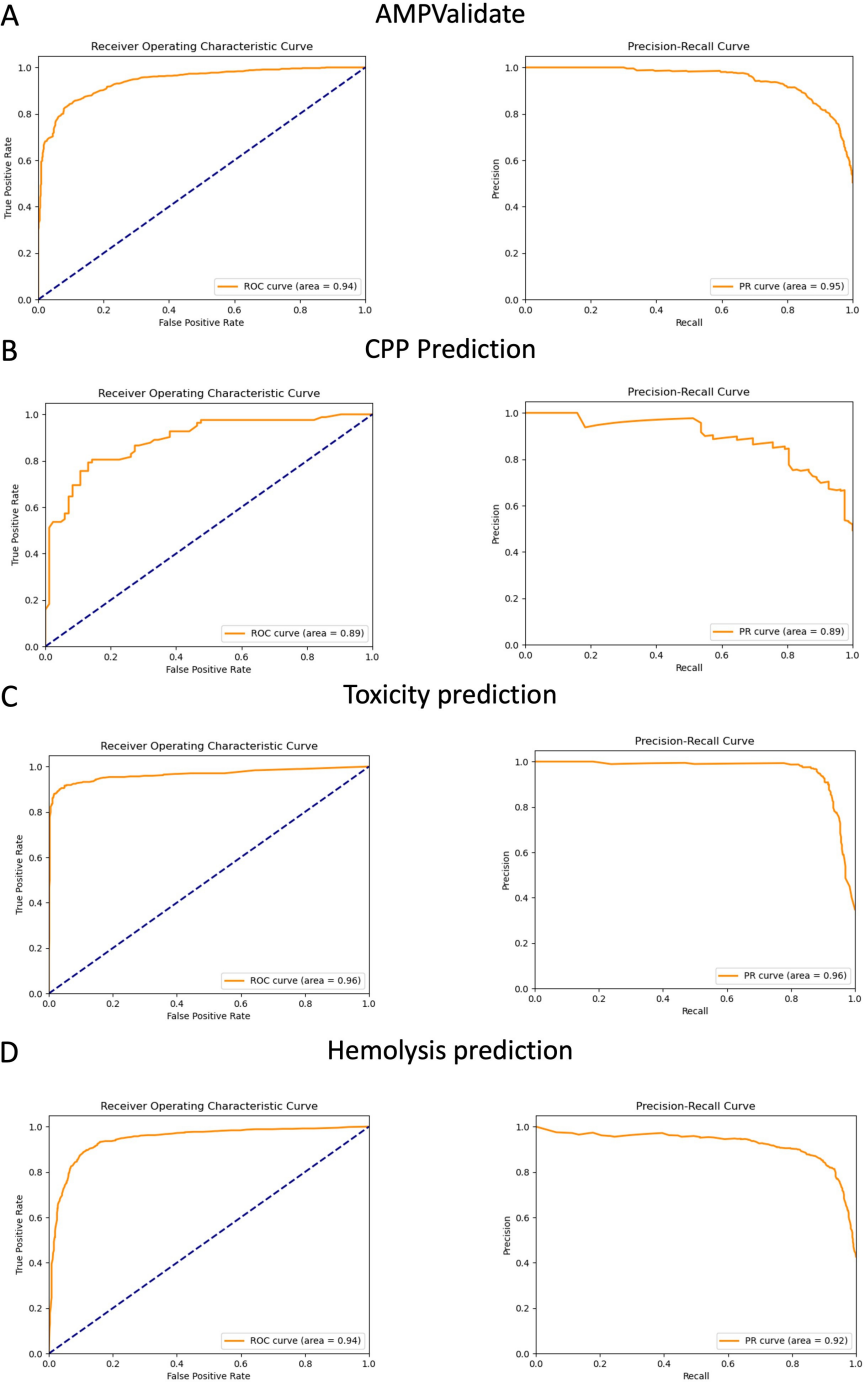
Receiver operating characteristic (ROC) curve cross-validation from peptide prediction random forest algorithms: AMPValidate (**A**), cell-penetrating capacity (**B**), peptide toxicity (**C**), and hemolysis (**D**).

AMPScreen is designed to identify regions in proteins with amino acid sequences compatible with AMP activity. However, a deeper analysis is advisable to establish whether the initial hits identified will exhibit ideal AMP behavior. To this end, the identified peptides are fed into AMPValidate, a random-forest-based classifier trained for this purpose. Based on the chosen AMP database, AMPValidate shows an accuracy of 0.87, an AUC of 0.94 and an MCC of 0.74, proving its ability to differentiate between “true” AMPs and other peptides with an accuracy similar to most AMP classifiers in the literature (Table 1; Fig. 2). We next used AMPScreen to scan the 20,398 reviewed sequences in the UniProt human proteome, and AMPA identified 41,964 peptides, 19,393 of which were considered potential AMPs after refinement with AMPValidate. This number of peptides identified by PyAMPA is comparable to other similar high-throughput studies (29). To experimentally validate the results, we selected and tested five peptides in the predicted human data set (Table S1). The peptides were selected from the human proteome based on four criteria: (i) a predicted probability of >0.5 by AMPValidate; (ii) a hemolysis probability of <0.5 by AMPSolve; (iii) no Cys residues, and (iv) size of 10–25 residues. From such predictions five peptides were thus selected to validate the method. All peptides showed broad activity against all strains tested, confirming PyAMPA’s effectiveness (Table 2). CD spectroscopy in aqueous solution and SDS micelles (Fig. S2, respectively) showed mostly random and β-sheet or α-helix structures, as typically observed for AMPs. The experimental peptides also showed satisfactory therapeutic windows, with low hemolysis and cytotoxicity levels relative to antimicrobial activity (Table 2).

**TABLE 1:** Evaluation values of the classifiers tested with the databases retrieved for generating each classifier: AMPValidate, and the random forest classifiers for the AMPSolve module (cell-penetrating capacity (CPC), toxicity and hemolysis)^*a*^

Property	Classifier	Accuracy	MCC
Antimicrobial activity	**AMPValidate^*b*^**	**0.87**	**0.74**
	*AI4AMP* (33)	*0.78*	*0.56*
	*AMPScanner* (34)	*0.80*	*0.61*
	*iAMPPred* (35)	*0.77*	*0.56*
Cell penetrating capacity	**AMPSolve (CPC**)	**0.89**	**0.77**
	*CellPPD* (36)	*0.88*	*0.77*
	*C2Pred* (37)	*0.80*	*0.61*
	*BChemRF-CPPred* (38)	*0.88*	*0.77*
Toxicity	**AMPSolve (toxicity**)	**0.94**	**0.88**
	*ToxinPred* (39)	*0.85*	*0.72*
	*ToxinPred3* (40)	*0.91*	*0.82*
	*PreTP-EL* (41)	*0.62*	*0.34*
Hemolysis	**AMPSolve (hemolysis**)	**0.88**	**0.76**
	*HemoPI-2* (42)	*0.77*	*0.52*
	*HemoPI-3* (42)	*0.73*	*0.46*
	*HemoPred* (43)	*0.72*	*0.44*

^*a*^ Values for accuracy, false positive rate (FPR), false negative rate (FNR) and the Matthew’s correlation coefficient (MCC) are shown. The performance of other classifiers with the same testing set is added for comparison.

^*b*^ Gray shading in the table is used to differentiate various predictions. Boldface is used to highlight the best pedictors and their respectives values.

**TABLE 2:** Minimal inhibitory concentration (MIC), hemolysis, and cytotoxicity of peptides

Peptide^*a*^	MIC (µM)							Toxicity	
	*E. coli*	*P. aeruginosa*	*A. baumanni*	*S. enterica*	*S. aureus*	*E. faecium*	*M. luteus*	*Hemolysis* ^*b*^	*Cytotoxicity^c^*
**1**	1.6	0.2	0.2	6	1.6	13	0.8	37	64
**2**	3.1	3.1	0.8	13	50	3.1	3.1	90	116
**3**	3.1	1.6	0.8	3.1	3.1	3.1	3.1	33	57
**4**	1.3	8	8	3.1	25	13	13	15	154
**5**	50	50	25	13	50	25	25	6.3	99

^*a*^ Sequences and additional data on the peptides are given in Table S1.

^*b*^ Measured as the percentage of hemolysis at 150 µM peptide concentration.

^*c*^ Measured as the IC_50_ in µM units in HepG2 cells.

### Calculation of AMP properties

As mentioned before, AMP usefulness depends on properties other than antimicrobial activity alone. To be effective as drugs, peptides must kill pathogens selectively, without damage to the host cells or toxicity for the organism. Also, they must remain in the body long enough to kill pathogens. To explore these issues, we trained models to predict hemolytic activity, toxicity and half-life in serum. All predictors were trained on specific databases using a random-forest classifier, except half-life, calculated by a multivariate regression model. Hemolytic activity and toxicity classifiers are suitable for peptides, with accuracies of 0.88 and 0.94 and MCCs of 0.76 and 0.88, respectively (Table 1; Fig. 2). A model was also trained to predict CPP behavior, namely the capacity to cross cell membranes and home on intracellular targets other than the membrane. This model also delivered successful predictions, with 0.89 accuracy and 0.77 MCC values (Table 1; Fig. 2). In all cases, all classifiers display excellent performance compared to other available tools (Table 1).

Finally, a model was trained to predict MICs toward bacteria by using activities measured on different strains, and developing a random forest classifier for each strain to predict the MIC. Again, the trained models showed substantial predictive ability (Table 3; Fig. S3). Predictions help define peptides suitable for a particular target (e.g., *S. aureus*) or a class (e.g., Gram-negative) of bacterial species.

**TABLE 3:** Antimicrobial activity predictor performance

Species	*R* ^2^
*S. aureus*	0.64
*B. subtilis*	0.63
*C. albicans*	0.52
*S. epidermidis*	0.63
*E. coli*	0.64
*E. faecalis*	0.26
*M. luteus*	0.49
*P. aeruginosa*	0.56
*S. typhimurium*	0.61
*K. pneumoniae*	0.72
*A. baumannii*	0.51

### Optimizing peptides with AMPMutate and AMPOptimize

**Fig 3:**
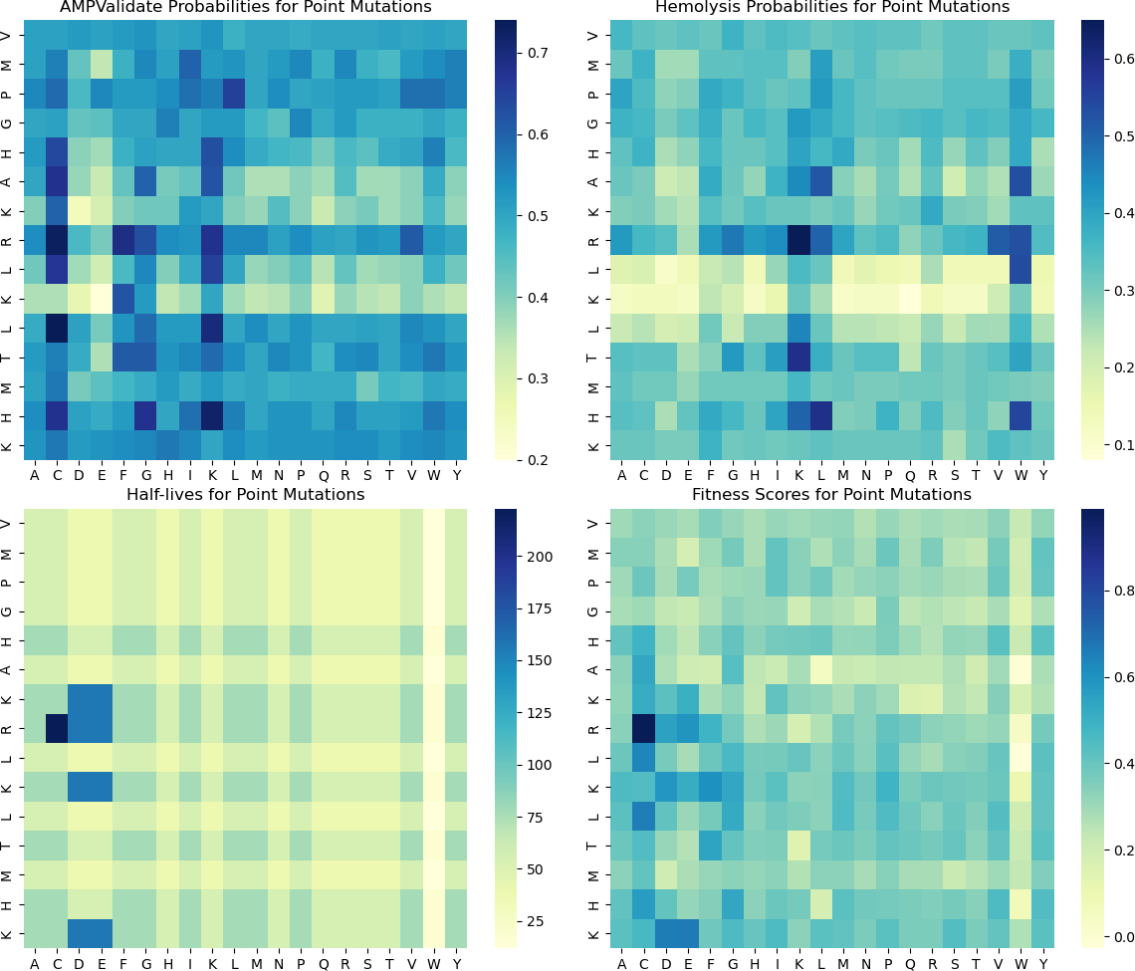
Single-point mutation fitness landscape on a peptide processed with AMPMutate. The original peptide sequence is displayed on the *y*-axis, and the individual mutations are indicated on the *x*-axis. Values in the heatmap are color-coded, with scales displayed to the right of each heatmap.

We have also developed two modules to refine initial hits into highly active AMPs by introducing mutations shedding light on the contribution of individual or groups of residues to AMP activity and properties. The first module, AMPMutate, generates all possible single-point mutations within a sequence, thus allowing to gauge the role of individual residues on AMP properties without disrupting activity. Properties in the mutant set, including MIC for all strains, are then evaluated by the AMPSolve module. AMPMutate next generates an Excel file with all mutants and their predicted properties, as well as heatmaps for antimicrobial activity, hemolytic activity, and half-life (Fig. 3).

**Fig 4:**
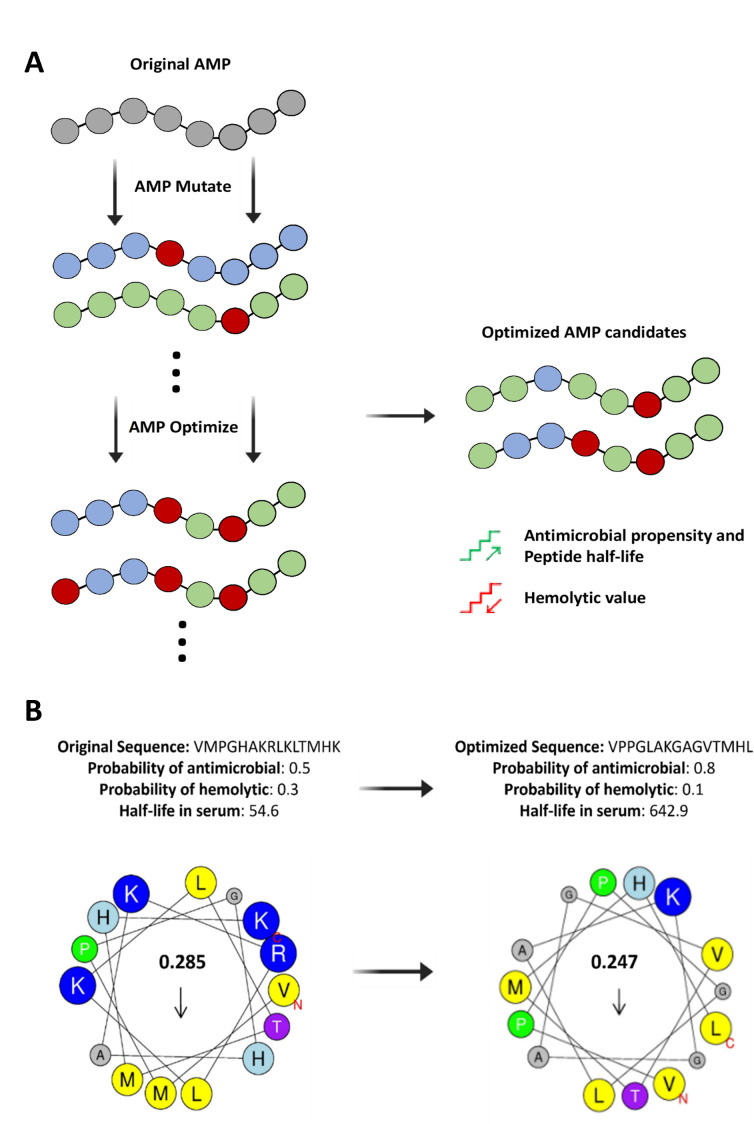
AMP optimization process. (**A**) Schematic workflow of the AMPOptimize module. Starting with a peptide query, AMPOptimize generates a library of mutated peptides which undergoes optimization by a genetic algorithm evaluating key AMP features: antimicrobial/hemolytic propensities and serum half-life. (**B**) Example of sequence optimization. The original sequence is improved in terms of antimicrobial propensity (higher), hemolytic probability (lower), and serum half-life (longer). These values, along with the corresponding sequences, are displayed at the top of panel (B). Number within the helical wheel is the calculated hydrophobic moment, with arrow indicating the direction. Subindexes C and N denote the sequence C-terminus and the N-terminus, respectively.

The second tool, AMPOptimize, is designed to identify the best AMPs for a given template. As already mentioned, AMPs detected in initial searches may have limited activity, be too toxic for practical clinical applications, or their protease stability may curtail druggability. From this starting point, AMPOptimize uses a genetic algorithm with a fitness function (Fig. 4A) designed to balance antimicrobial propensity, hemolytic activity, and half-life. After several AMPOptimize cycles, a plateau sequence with optimal scores on all three criteria is reached (Fig. 4B). Users can access the script and modify the fitness function to reward or penalize properties according to their goals.

### Conclusion

PyAMPA is a novel bioinformatic platform enabling discovery of AMP leads through five modules: AMPScreen, AMPValidate, AMPSolve, AMPMutate, and AMPOptimize. Each module is used to generate various candidates during an AMP discovery course. The combined use of the five algorithms, particularly the point-mutation and genetic optimization modules, makes PyAMPA a robust and efficient tool for high-throughput screening of proteomes, unveiling putative AMP motifs and improving their eventual druggability.

## Data Availability

The data sets and code used in this project are available at https://github.com/SysBioUAB/PyAMPA.

## SUPPLEMENTAL MATERIAL

Supplemental Material msystems.01358-23-s0001.pdf Supplemental figures and table.

ASM does not own the copyrights to Supplemental Material that may be linked to, or accessed through, an article. The authors have granted ASM a non-exclusive, world-wide license to publish the Supplemental Material files. Please contact the corresponding author directly for reuse.

## References

[1] Levy SB, Marshall B. 2004. Antibacterial resistance worldwide: causes, challenges and responses. Nat Med 10:S122–S129. doi:10.1038/nm1145 15577930

[2] Jones KE, Patel NG, Levy MA, Storeygard A, Balk D, Gittleman JL, Daszak P. 2008. Global trends in emerging infectious diseases. Nature 451:990–993. doi:10.1038/nature06536 18288193 PMC5960580

[3] Cantas L, Shah SQA, Cavaco LM, Manaia CM, Walsh F, Popowska M, Garelick H, Bürgmann H, Sørum H. 2013. A brief multi-disciplinary review on antimicrobial resistance in medicine and its linkage to the global environmental microbiota. Front Microbiol 4:96. doi:10.3389/fmicb.2013.00096 23675371 PMC3653125

[4] Laxminarayan R, Matsoso P, Pant S, Brower C, Røttingen J-A, Klugman K, Davies S. 2016. Access to effective antimicrobials: a worldwide challenge. Lancet 387:168–175. doi:10.1016/S0140-6736(15)00474-2 26603918

[5] Van Boeckel TP, Pires J, Silvester R, Zhao C, Song J, Criscuolo NG, Gilbert M, Bonhoeffer S, Laxminarayan R. 2019. Global trends in antimicrobial resistance in animals in low- and middle-income countries. Science 365:eaaw1944. doi:10.1126/science.aaw1944 31604207

[6] Christaki E, Marcou M, Tofarides A. 2020. Antimicrobial resistance in bacteria: mechanisms, evolution, and persistence. J Mol Evol 88:26–40. doi:10.1007/s00239-019-09914-3 31659373

[7] Brown ED, Wright GD. 2016. Antibacterial drug discovery in the resistance era. Nature 529:336–343. doi:10.1038/nature17042 26791724

[8] Hancock REW, Sahl H-G. 2006. Antimicrobial and host-defense peptides as new anti-infective therapeutic strategies. Nat Biotechnol 24:1551–1557. doi:10.1038/nbt1267 17160061

[9] Mahlapuu M, Håkansson J, Ringstad L, Björn C. 2016. Antimicrobial peptides: an emerging category of therapeutic agents. Front Cell Infect Microbiol 6:194. doi:10.3389/fcimb.2016.00194 28083516 PMC5186781

[10] Torrent M, Di Tommaso P, Pulido D, Nogués MV, Notredame C, Boix E, Andreu D. 2012. AMPA: an automated web server for prediction of protein antimicrobial regions. Bioinformatics 28:130–131. doi:10.1093/bioinformatics/btr604 22053077

[11] Dee W. 2022. LMPred: predicting antimicrobial peptides using pre-trained language models and deep learning. Bioinform Adv 2:vbac021. doi:10.1093/bioadv/vbac021 36699381 PMC9710646

[12] Li C, Sutherland D, Hammond SA, Yang C, Taho F, Bergman L, Houston S, Warren RL, Wong T, Hoang LMN, Cameron CE, Helbing CC, Birol I. 2022. AMPlify: attentive deep learning model for discovery of novel antimicrobial peptides effective against WHO priority pathogens. BMC Genomics 23:77. doi:10.1186/s12864-022-08310-4 35078402 PMC8788131

[13] Lee H, Lee S, Lee I, Nam H. 2023. AMP-BERT: prediction of antimicrobial peptide function based on a BERT model. Protein Sci 32:e4529. doi:10.1002/pro.4529 36461699 PMC9793967

[14] Yan K, Lv H, Guo Y, Peng W, Liu B. 2023. sAMPpred-GAT: prediction of antimicrobial peptide by graph attention network and predicted peptide structure. Bioinformatics 39:btac715. doi:10.1093/bioinformatics/btac715 36342186 PMC9805557

[15] Lin T-T, Yang L-Y, Lu I-H, Cheng W-C, Hsu Z-R, Chen S-H, Lin C-Y. 2021. AI4AMP: an antimicrobial peptide predictor using physicochemical property-based encoding method and deep learning. mSystems 6:e0029921. doi:10.1128/mSystems.00299-21 34783578 PMC8594441

[16] Hilpert K, Volkmer-Engert R, Walter T, Hancock REW. 2005. High-throughput generation of small antibacterial peptides with improved activity. Nat Biotechnol 23:1008–1012. doi:10.1038/nbt1113 16041366

[17] Andreu D, Torrent M. 2015. Prediction of bioactive peptides using artificial neural networks. Methods Mol Biol 1260:101–118. doi:10.1007/978-1-4939-2239-0_7 25502378

[18] Jukič M, Bren U. 2022. Machine learning in antibacterial drug design. Front Pharmacol 13:864412. doi:10.3389/fphar.2022.864412 35592425 PMC9110924

[19] Sowers A, Wang G, Xing M, Li B. 2023. Advances in antimicrobial peptide discovery via machine learning and delivery via nanotechnology. Microorganisms 11:1129. doi:10.3390/microorganisms11051129 37317103 PMC10223199

[20] Briard B, Karki R, Malireddi RKS, Bhattacharya A, Place DE, Mavuluri J, Peters JL, Vogel P, Yamamoto M, Kanneganti T-D. 2019. Fungal ligands released by innate immune effectors promote inflammasome activation during Aspergillus fumigatus infection. Nat Microbiol 4:316–327. doi:10.1038/s41564-018-0298-0 30510167 PMC6619501

[21] Oyama LB, Girdwood SE, Cookson AR, Fernandez-Fuentes N, Privé F, Vallin HE, Wilkinson TJ, Golyshin PN, Golyshina OV, Mikut R, Hilpert K, Richards J, Wootton M, Edwards JE, Maresca M, Perrier J, Lundy FT, Luo Y, Zhou M, Hess M, Mantovani HC, Creevey CJ, Huws SA. 2017. The rumen microbiome: an underexplored resource for novel antimicrobial discovery. NPJ Biofilms Microbiomes 3:33. doi:10.1038/s41522-017-0042-1 29214045 PMC5711939

[22] Man SM, Karki R, Sasai M, Place DE, Kesavardhana S, Temirov J, Frase S, Zhu Q, Malireddi RKS, Kuriakose T, Peters JL, Neale G, Brown SA, Yamamoto M, Kanneganti T-D. 2016. IRGB10 liberates bacterial ligands for sensing by the AIM2 and caspase-11-NLRP3 inflammasomes. Cell 167:382–396. doi:10.1016/j.cell.2016.09.012 27693356 PMC5074697

[23] Sandín D, Valle J, Chaves-Arquero B, Prats-Ejarque G, Larrosa MN, González-López JJ, Jiménez MÁ, Boix E, Andreu D, Torrent M. 2021. Rationally modified antimicrobial peptides from the N-terminal domain of human RNase 3 show exceptional serum stability. J Med Chem 64:11472–11482. doi:10.1021/acs.jmedchem.1c00795 34342438 PMC8483441

[24] Torrent M, Pulido D, de la Torre BG, García-Mayoral MF, Nogués MV, Bruix M, Andreu D, Boix E. 2011. Refining the eosinophil cationic protein antibacterial pharmacophore by rational structure minimization. J Med Chem 54:5237–5244. doi:10.1021/jm200701g 21696142

[25] Pandey P, Patel V, George NV, Mallajosyula SS. 2018. KELM-CPPpred: kernel extreme learning machine based prediction model for cell-penetrating peptides. J Proteome Res 17:3214–3222. doi:10.1021/acs.jproteome.8b00322 30032609

[26] Salem M, Keshavarzi Arshadi A, Yuan JS. 2022. AMPDeep: hemolytic activity prediction of antimicrobial peptides using transfer learning. BMC Bioinformatics 23:389. doi:10.1186/s12859-022-04952-z 36163001 PMC9511757

[27] Gupta S, Kapoor P, Chaudhary K, Gautam A, Kumar R, Raghava GPS, Open Source Drug Discovery Consortium. 2013. In silico approach for predicting toxicity of peptides and proteins. PLoS One 8:e73957. doi:10.1371/journal.pone.0073957 24058508 PMC3772798

[28] Witten J, Witten Z. 2019. Deep learning regression model for antimicrobial peptide design. bioRxiv. doi:10.1101/692681

[29] Torrent M, Nogués VM, Boix E. 2009. A theoretical approach to spot active regions in antimicrobial proteins. BMC Bioinformatics 10:373. doi:10.1186/1471-2105-10-373 19906288 PMC2780422

[30] Cavaco M, Valle J, Flores I, Andreu D, A R B Castanho M. 2021. Estimating peptide half-life in serum from tunable, sequence-related physicochemical properties. Clin Transl Sci 14:1349–1358. doi:10.1111/cts.12985 33641212 PMC8301568

[31] Wiegand I, Hilpert K, Hancock REW. 2008. Agar and broth dilution methods to determine the minimal inhibitory concentration (MIC) of antimicrobial substances. Nat Protoc 3:163–175. doi:10.1038/nprot.2007.521 18274517

[32] Liu H, Lei M, Du X, Cui P, Zhang S. 2015. Identification of a novel antimicrobial peptide from amphioxus Branchiostoma japonicum by in silico and functional analyses. Sci Rep 5:18355. doi:10.1038/srep18355 26680226 PMC4683396

[33] Lin T-T, Yang L-Y, Lu I-H, Cheng W-C, Hsu Z-R, Chen S-H, Lin C-Y. 2021. AI4AMP: an antimicrobial peptide predictor using physicochemical property-based encoding method and deep learning. mSystems 6:e0029921. doi:10.1128/mSystems.00299-21 34783578 PMC8594441

[34] Veltri D, Kamath U, Shehu A. 2018. Deep learning improves antimicrobial peptide recognition. Bioinformatics 34:2740–2747. doi:10.1093/bioinformatics/bty179 29590297 PMC6084614

[35] Meher PK, Sahu TK, Saini V, Rao AR. 2017. Predicting antimicrobial peptides with improved accuracy by incorporating the compositional, physico-chemical and structural features into Chou's general PseAAC. Sci Rep 7:42362. doi:10.1038/srep42362 28205576 PMC5304217

[36] Gautam A, Chaudhary K, Kumar R, Sharma A, Kapoor P, Tyagi A, Raghava GPS, Open source drug discovery consortium. 2013. In silico approaches for designing highly effective cell penetrating peptides. J Transl Med 11:74. doi:10.1186/1479-5876-11-74 23517638 PMC3615965

[37] Tang H, Su Z-D, Wei H-H, Chen W, Lin H. 2016. Prediction of cell-penetrating peptides with feature selection techniques. Biochem Biophys Res Commun 477:150–154. doi:10.1016/j.bbrc.2016.06.035 27291150

[38] de Oliveira ECL, Santana K, Josino L, Lima E Lima AH, de Souza de Sales Júnior C. 2021. Predicting cell-penetrating peptides using machine learning algorithms and navigating in their chemical space. Sci Rep 11:7628. doi:10.1038/s41598-021-87134-w 33828175 PMC8027643

[39] Gupta S, Kapoor P, Chaudhary K, Gautam A, Kumar R, Raghava GPS, Open Source Drug Discovery Consortium. 2013. In silico approach for predicting toxicity of peptides and proteins. PLoS One 8:e73957. doi:10.1371/journal.pone.0073957 24058508 PMC3772798

[40] Rathore AS, Arora A, Choudhury S, Tijare P, Raghava GPS. 2023. ToxinPred 3.0: an improved method for predicting the toxicity of peptides. bioRxiv. doi:10.1101/2023.08.11.552911 39038391

[41] Guo Y, Yan K, Lv H, Liu B. 2021. PreTP-EL: prediction of therapeutic peptides based on ensemble learning. Brief Bioinformatics 22. doi:10.1093/bib/bbab358 34459488

[42] Chaudhary K, Kumar R, Singh S, Tuknait A, Gautam A, Mathur D, Anand P, Varshney GC, Raghava GPS. 2016. A web server and mobile app for computing hemolytic potency of peptides. Sci Rep 6:22843. doi:10.1038/srep22843 26953092 PMC4782144

[43] Win TS, Malik AA, Prachayasittikul V, S Wikberg JE, Nantasenamat C, Shoombuatong W. 2017. HemoPred: a web server for predicting the hemolytic activity of peptides. Future Med Chem 9:275–291. doi:10.4155/fmc-2016-0188 28211294

