# Supplementary material for: PyAMPA: a high-throughput prediction and optimization tool for antimicrobial peptides
Source: mSystems. 2024 Jun 27;9(7):e01358-23. doi: 10.1128/msystems.01358-23 (PMC11264690; doi:10.1128/msystems.01358-23)
Supplement: Supplemental Material — Supplemental figures and table. [file msystems.01358-23-s0001.pdf]

Supplementary Information

for

PyAMPA: a high-throughput prediction and optimization tool for antimicrobial peptides

Marc Ramos-Llorens[1‡], Roberto Bello-Madruga [1], Javier Valle[2], David Andreu [2,*], and Marc Torrent[1,*]

[1] Systems Biology of Infection Lab, Department of Biochemistry and Molecular Biology, Biosciences Faculty, Universitat Autònoma de Barcelona, 08193 Cerdanyola del Vallès, Spain.

[2] Barcelona Biomedical Research Park, Department of Medicine and Life Sciences, Universitat Pompeu Fabra, 08003 Barcelona, Spain

[‡] Current address: Instituto de Acuicultura Torre de la Sal, Consejo Superior de Investigaciones Científicas (IATS-CSIC), 12595 Ribera de Cabanes, Castellón, Spain

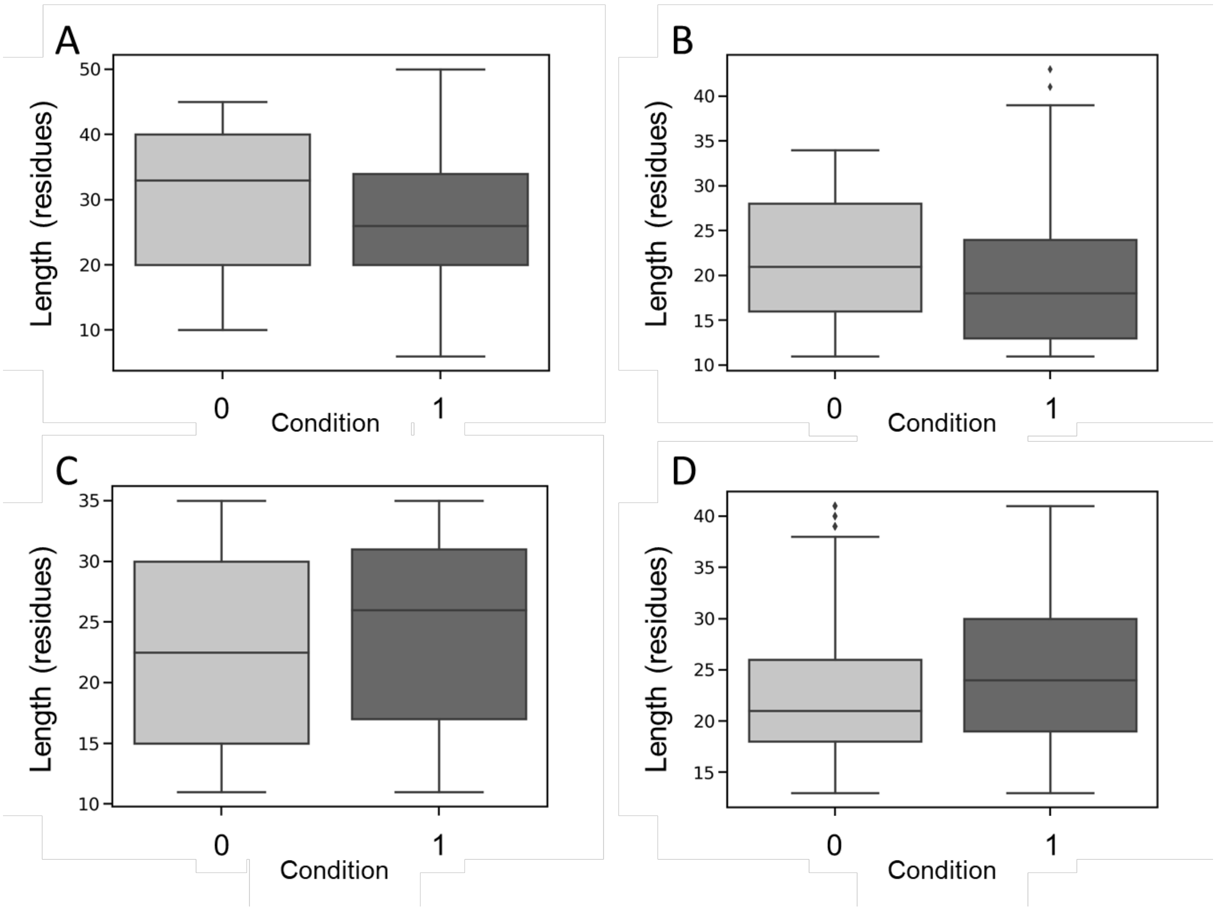

Supplementary Figure 1. Distribution of peptide length in amino acids for the datasets tested. Boxplots represent the peptide length distribution for the datasets used to predict the following properties: (A) antimicrobial nature, (B) cell-penetrating capacity, (C) peptide toxicity and (D) hemolysis. Condition represents positive (1) or negative (0) entries for each dataset used for peptide quality prediction. No significant differences were observed in any of the properties analyzed ($p > 0.05$, Mann–Whitney–Wilcoxon test).

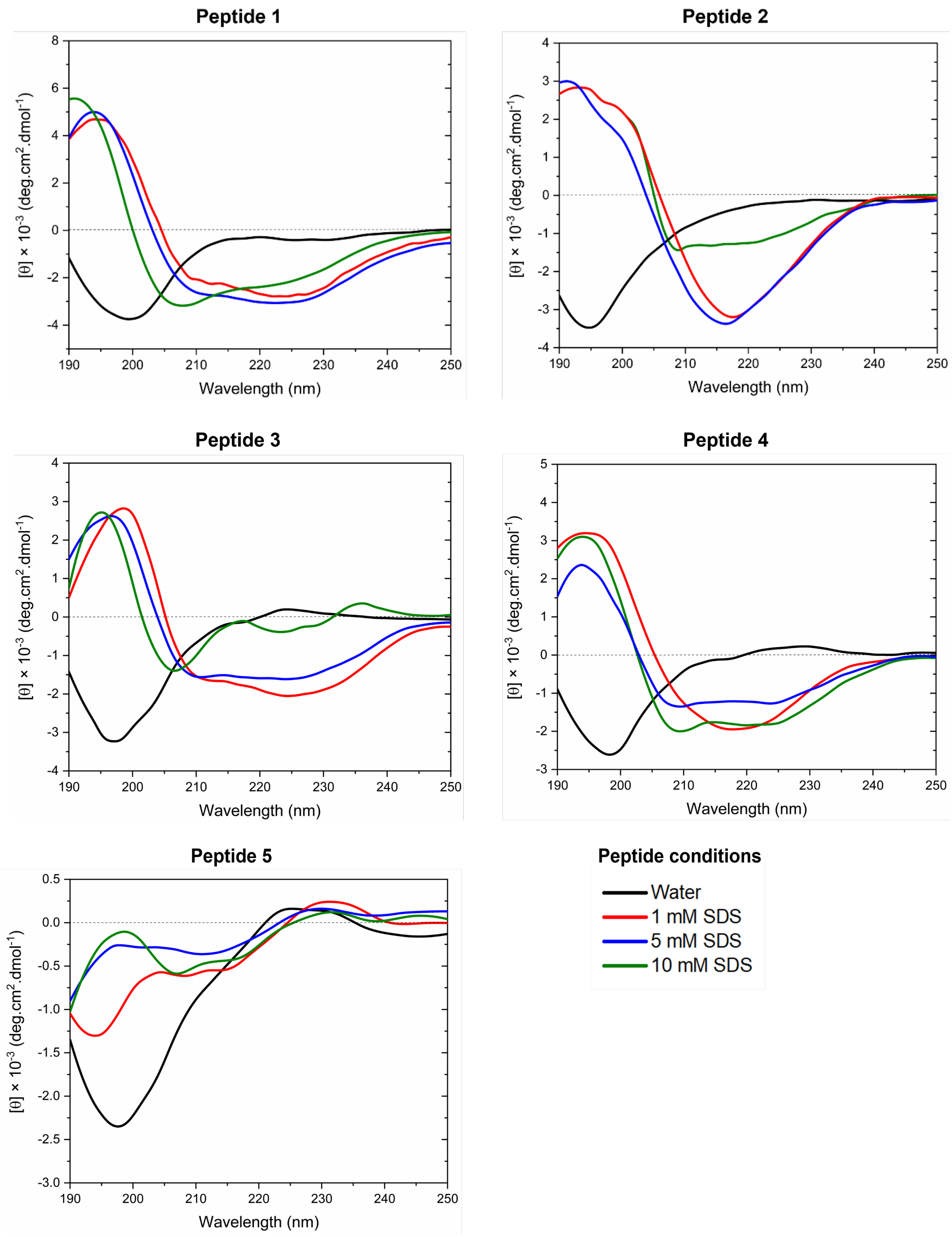
Peptide 1
Peptide 2
Peptide 3
Peptide 4
Peptide 5
[θ] × 10^{-3} (deg.cm^2.dmol^{-1})
Wavelength (nm)
190
200
210
220
230
240
250
Peptide conditions
Water
1 mM SDS
5 mM SDS
10 mM SDS

Supplementary Figure 2. Circular Dichroism Analysis of peptides. Peptides (1-5) were tested for secondary structure in water and in membrane-like environment with SDS monomers and micelles, respectively.

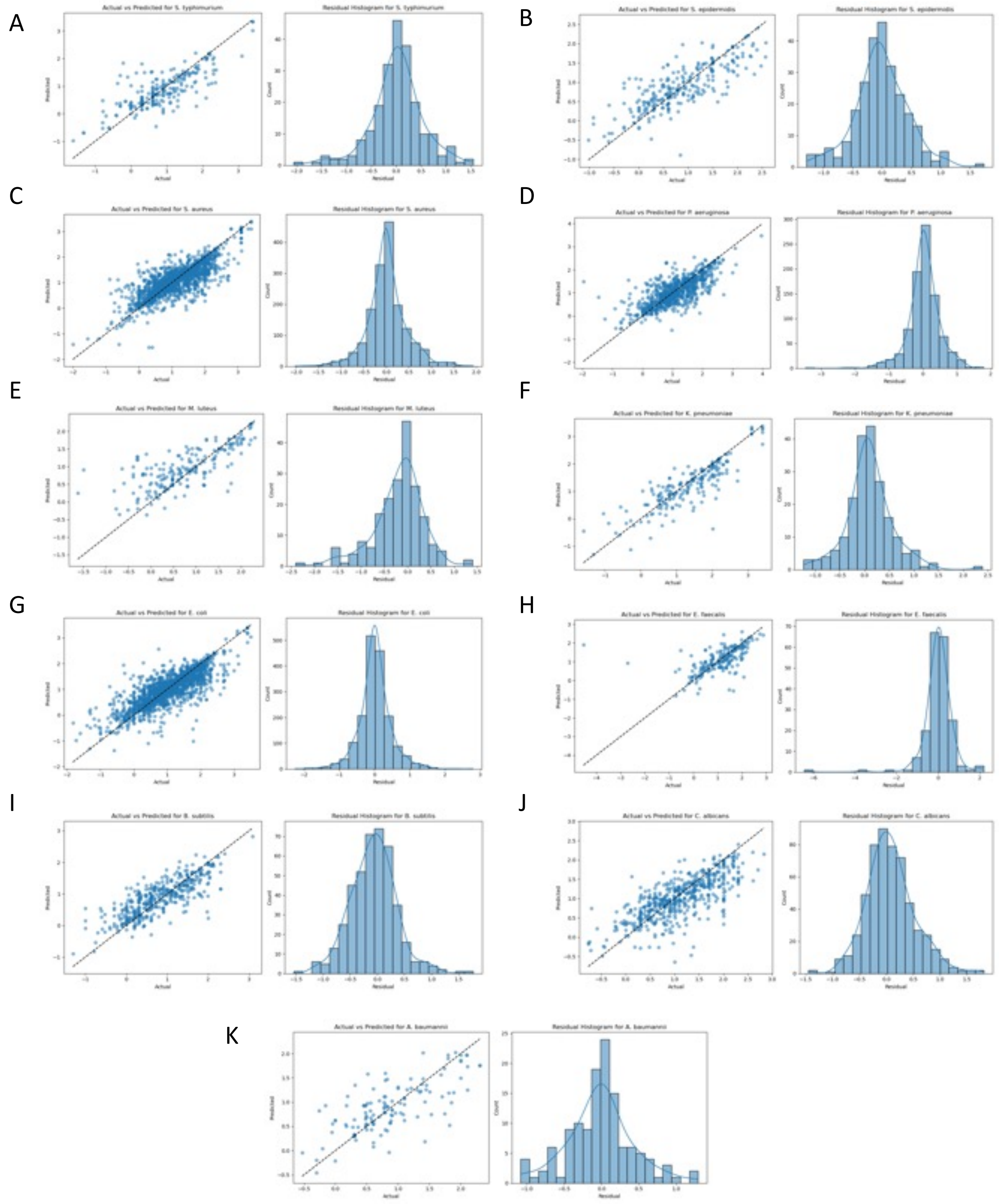

Supplementary Figure 3. Regression and residual plot for antimicrobial activity prediction. Each panel represents a unique bacterial strain: (A) *S. typhimurium,* (B) *S. epidermidis*, (C) *S. aureus*, (D) *P. aeruginosa*, (E) *M. luteus*, (F) *K. pneumoniae*, (G) *E. coli*, (H) *E. faecalis*, (I) *B. subtilis,* (J) *A. baumannii*, (K) *C. albicans*. In the regression plot, the x-axis represents the

predicted value for minimal inhibitory concentrations (MIC) and the y-axis the real MIC retrieved from the GRAMPA database.

Supplementary Table 1.

Peptide	Sequence [a]	Derived from UniProt ID	Residues	MW (Da) calc / found
**1**	LGIGLFMRRRHIVRKRTLRRLLQE	Q504U8	24	3017.70 / 3017.35
**2**	KKLKRYFVDYRRVLV	Q9H4K7	15	1982.42 / 1982.40
**3**	AGAKRYKYLRRLFRFR	Q53HI1	16	2100.52 / 2100.50
**4**	LVRAYVQRYRYFRKHGRK	Q9H7N4	18	2395.82 / 2395.80
**5**	YRRFLRPRYRVAY	Q9Y6C2	13	1815.13 / 1815.2

[a] C-terminus amidated
